# Myxosporean hyperparasites of gill monogeneans are basal to the Multivalvulida

**DOI:** 10.1186/1756-3305-4-220

**Published:** 2011-11-24

**Authors:** Mark A Freeman, Andrew P Shinn

**Affiliations:** 1 Institute of Biological Sciences & Institute of Ocean and Earth Sciences, University of Malaya, Kuala Lumpur, 50603 Malaysia; 2 Institute of Aquaculture, University of Stirling, Stirling FK9 4LA, UK

## Abstract

**Background:**

Myxosporeans are known from aquatic annelids but parasitism of platyhelminths by myxosporeans has not been widely reported. Hyperparasitism of gill monogeneans by *Myxidium giardi *has been reported from the European eel and *Myxidium*-like hyperparasites have also been observed during studies of gill monogeneans from Malaysia and Japan.

The present study aimed to collect new hyperparasite material from Malaysia for morphological and molecular descriptions. In addition, PCR screening of host fish was undertaken to determine whether they are also hosts for the myxosporean.

**Results:**

Heavy myxosporean infections were observed in monogeneans from two out of 14 fish and were detected from a further five fish using specific PCRs and pooled monogenean DNA. Positive DNA isolates were sequenced and were from a single species of myxosporean. Myxospore morphology was consistent with *Myxidium *with histozoic development in the parenchymal tissues of the monogenean. Simultaneous infections in the fish could not be confirmed microscopically; however, identical myxosporean DNA could be amplified from kidney, spleen and intestinal tract tissues using the specific PCR. Small subunit (SSU) rDNA for the myxosporean was amplified and was found to be most similar (92%) to that of another hyperparasitic myxosporean from a gill monogenean from Japan and to numerous multivalvulidan myxosporeans from the genus *Kudoa *(89-91%). Phylogenetic analyses placed the hyperparasite sequence basally to clades containing *Kudoa*, *Unicapsula *and *Sphaerospora*.

**Conclusions:**

The myxosporean infecting the gill monogenean, *Diplectanocotyla gracilis*, from the Indo-Pacific tarpon, *Megalops cyprinoides*, is described as a new species, *Myxidium incomptavermi*, based on a histozoic development in the monogenean host and its phylogenetic placement.

We have demonstrated for the first time that a myxosporean hyperparasite of gill monogeneans is detectable in the fish host. However, myxospores could not be isolated from the fish and confirmation was by PCR alone. The relationship between the myxosporean infection in gill monogeneans and the presence of parasitic DNA in fish is not yet fully understood. Nonetheless, myxospores with a *Myxidium*-like morphology, two of which we have shown to be phylogenetically related, have now been reported to develop in three different gill monogeneans, indicating that myxosporeans are true parasites of monogeneans.

## Background

Myxosporean parasites are commonly found infecting numerous fish species, proliferating in different target organs and tissues where distinctive myxospores develop. Known myxosporean life cycles involve a second obligate host, typically a free-living annelid worm, where morphologically dissimilar actinospores are produced, which are infectious to the fish host, thus completing the life cycle. Myxosporeans are also known to infect non-fish vertebrate hosts such as amphibians, reptiles, waterfowl and insectivorous mammals [1-4], all of which result in characteristic myxospore production, but whose life cycles are unknown. A single report has shown the myxospore phase of development in an invertebrate, where *Kudoa *sp. was found in the muscles of the arm of the giant octopus *Paroctopus dofleini *[5]. However, myxospore development has never been reported from annelid hosts and only actinospores are produced.

*Myxidium *Bütschli, 1882 is a large genus of over 200 mostly coelozoic myxosporeans, infecting various vertebrate hosts such as fish, amphibians and reptiles [6]. They have a polyphyletic distribution within myxosporean phylogenetics [4], but are not currently represented in the multivalvulidan clade. Multivalvulidan myxosporeans represent the marine order Multivalvulida Shulman, 1959 that are predominantly histozoic and infect the musculature of marine fishes [6]. Whipps et al. [7] used rDNA sequence data and simplified the group, which currently contains two families, Trilosporidae Shulman, 1959 and Kudoidae Meglitsch, 1960. The Trilosporidae have three polar capsules and three shell valves and are considered to be the basal group of the order. The Kudoidae now accommodate all marine myxosporeans with four or more shell valves and polar capsules and contain over 70 described species. The multivalvulidans are important parasites that can have a considerable negative impact on the condition of farmed marine finfish and commercial fisheries [8]. *Kudoa *spp. have also been linked to, and may be the cause of, gastroallergic symptoms in humans after ingestion of *Kudoa*-infected fish products in Europe and Japan [9,10]. The multivalvulidans are the only large group of myxosporeans for which no life cycle or definitive host data is available.

Hyperparasitism of platyhelminths by myxosporeans is not common. Myxosporeans from the genus *Fabespora *have been reported from the tegument and parenchyma of digenean trematodes [11,12]. Hyperparasitism by *Myxidium giardi *has also been reported in the gill monogenean *Pseudodactylogyrus bini *from the European eel *Anguilla anguilla *[13]. Myxosporean hyperparasites have also been observed during studies of the gill monogeneans from coastal fish in Malaysia and Japan. However, infections by these myxosporeans only became apparent after samples had been permanently mounted; therefore complete descriptions of the spores were not possible [14]. In all the above cases, platyhelminths are parasitic in brackish water fish, serious pathology results in the monogeneans and the myxosporean infections cannot be detected in the host fish.

The Indo-Pacific tarpon, *Megalops cyprinoides *(Broussonet, 1782), (Order Elopiformes: tarpons and tenpounders) inhabits tropical coastal and brackish waters of the Indo-Pacific, migrating between the open sea and inland rivers and mangroves [15]. Elopiformes are related to, but do not resemble, eels and like eels, they spawn at sea producing leptocephalic larvae that later migrate inland [16]. *Megalops cyprinoides *are frequently netted in the mangroves and coastal waters of Malaysia, commonly 20-30 cm in length, but are not highly prized as food fish.

In the present study, gill monogeneans from *M. cyprinoides *are examined for the presence of hyperparasitic myxosporeans in order to complete morphological descriptions and DNA analyses.

## Methods

### Collection and sampling of fish and Monogenea

*Megalops cyprinoides *were collected directly from local fishermen and from fish markets on Langkawi Island, Peninsular Malaysia. Fish were examined fresh on the day of purchase. Gill arches were removed and viewed with a dissecting microscope (Olympus SZ4045), monogenean parasites were isolated with forceps and a fine glass pipette, slide-mounted with a coverslip and viewed using a portable phase contrast compound microscope (Nikon YS2-H). Visibly infected monogeneans (those confirmed to have myxospores present internally) were either removed from the slide and used for individual DNA extraction or disrupted by squashing to release fresh myxospores for photography. Visibly uninfected monogeneans were removed from the slides, pooled for each fish, and fixed in 95% ethanol for DNA analysis. Digital images of infected monogeneans and fresh myxospores were taken in the field using a Dino-eye microscope eye-piece camera (DinoLite, Dutech Scientific, Malaysia) and DinoCapture software version 3.3.0.16 (AnMo Electronics Corporation, Taiwan). Myxospores were described following the guidelines of Lom & Arthur [17]. After photography of released myxospores, cover slips were removed, slides air dried and later stained with Diff-Quik^®^. Due to time constraints, some gill monogeneans from heavily infected fish were not all individually observed with the compound microscope. Instead, they were removed from the gills, a sub-sample used for microscopy, the remainder pooled from each fish and fixed in 95% ethanol for the DNA analysis. During the monogenean removal process each gill arch was carefully examined for the presence of myxosporean gill cysts and signs of other parasitic infections, and a sample of gill tissue was taken for the DNA analysis. During a final sampling trip, a fish confirmed as having a gill monogenean with a visible myxosporean infection (fish 2 see below) was dissected and numerous tissue samples (gill, kidney, gall bladder, bile, liver, spleen, intestine, stomach, heart and brain) were taken for the DNA analysis.

All fish were also fully dissected and examined for gross signs of infection or abnormalities in the internal organs. In addition, the kidney, gall bladder and contents, intestine, brain, heart and chamber blood, liver and spleen were carefully investigated for the presence of myxosporean infections using tissue stamps or squashes and phase contrast microscopy.

### DNA preparation and analyses

DNA extractions were performed using a GeneMATRIX DNA extraction kit (EURx Poland) following the manufacturer's tissue protocol. Myxosporean SSU rDNA was amplified using the universal primer 18e [18] and the myxosporean primers and PCR conditions described by Freeman et al. [19] with the temperature profile: initial denaturation 95°C for 4 min, 35 cycles of denaturation 94°C for 30 sec, annealing 55°C for 45 sec, extension 72°C for 1 min, followed by a 7 min terminal extension at 72°C. Additional specific primers, Bu-F 5' GGT CAA TGT ATT TGG ACG TCG 3' and Bu-R 5' TGA CTC CGG TTG TCT CTC TAA G 3' were designed for the hyperparasitic myxosporean from the initial sequence reads, in order to amplify 720 bp of the SSU using the same PCR conditions as described above. DNA was also amplified from an infected monogenean; using the primer pair C1 and D2 [20] to amplify the D1-D2 domains of the large subunit ribosomal DNA (LSU rDNA) to assist in any future studies with the molecular identification of infected gill monogeneans.

DNA sequencing was conducted using BigDyeTM Terminator Cycle Sequencing chemistry utilising the same oligonucleotide primers that were used for the original PCRs and was performed on all PCR positive samples. Both directions of each amplicon were sequenced for all products and compared to sequences available in the GenBank databases using nucleotide-nucleotide BLAST searches [21] to verify a myxosporean origin. Contiguous sequences were constructed manually using CLUSTAL_X [22] and BioEdit [23]. CLUSTAL_X [22] was used for the alignment of the SSU rDNA sequences of 57 myxozoan taxa (see additional file 1, S1) with the settings for gap opening/extension penalties being adjusted manually to achieve optimum alignments. Regions of ambiguous sequence alignments were manually edited using the BioEdit sequence alignment editor [23].

Phylogenetic analyses were performed using the maximum likelihood methodology in PhyML [24] with the general time-reversible (GTR) substitution model selected (gamma shape parameter = 0.520 and the proportion of invariant = 0.128) and 100 bootstrap repeats, and Bayesian inference (BI) analyses using MrBayes v. 3.0 [25]. Models of nucleotide substitution were evaluated for the data using MrModeltest v. 2.2 [26]. The most parameter-rich evolutionary model based on the AIC was the GTR+I+G model of evolution. Therefore, the settings used for the analysis were nst = 6, with the gamma-distributed rate variation across sites and a proportion of invariable sites (rates = invgamma) with optimal parameters estimated from the data (gamma shape parameter uniformly distributed on the interval (0.00, 200.00), with the proportion of invariable sites uniformly distributed on the interval (0.00, 1.00) and gamma distribution approximated using 4 categories). The priors on state frequency were left at the default setting (Prset statefreqpr = dirichlet (1,1,1,1)). Posterior probability distributions were generated using the Markov Chain Monte Carlo (MCMC) method with four chains being run simultaneously for 1,000,000 generations. Burn in was set at 2500 and trees were sampled every 100 generations making a total of 7500 trees used to compile the majority rule consensus trees.

In accordance with section 8.6 of the ICZN's International Code of Zoological Nomenclature, copies of this article are deposited at the following five publicly accessible libraries: Natural History Museum, London, UK; American Museum of Natural History, New York, USA; Museum National d'Histoire Naturelle, Paris, France; Russian Academy of Sciences, Moscow, Russia; Academia Sinica, Taipei, Taiwan.

## Results

Gill monogeneans were collected from fourteen *M. cyprinoides *ranging in size from 12 to 32.5 cm in fork length (FL). The number of monogeneans collected from each fish ranged from 3 to over 100, twelve additional fish had no gill monogeneans present. Two individual monogeneans, from different fish (subsequently referred to as: fish 1 FL 23 cm, total of 20 monogeneans collected; fish 2 FL 32.5 cm, total of 50 monogeneans collected), could be detected with visible myxosporean infections. One was removed (fish 1) intact for DNA analysis and the other (fish 2) disrupted to release myxospores for photography and later stained for submission as type material.

### Identification of monogenean host

**Figure 1:**
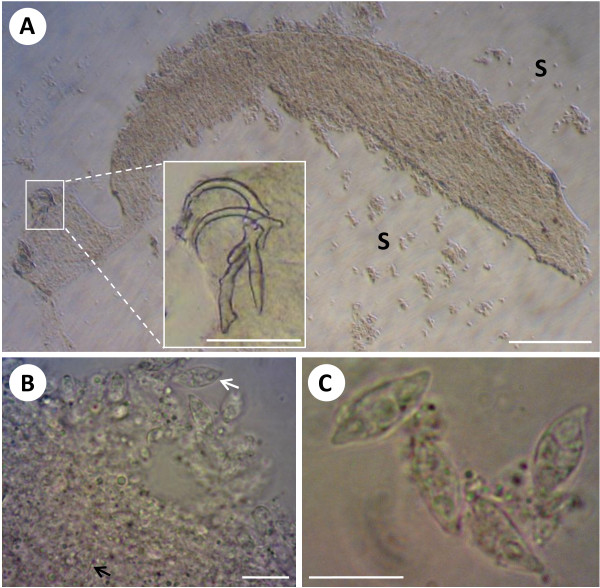
**Histozoic myxosporean, *Myxidium incomptavermi *n. sp., infecting the gill monogenean *Diplectanocotyla gracilis***. A) Flattened monogenean showing no internal organ definition, with myxospores (S) released from the body cavity, bar = 200 μm. Inset) fine detail of the haptoral sclerites, bar = 50 μm. B) spores released from ruptured parenchymal tissues (white arrow) with numerous spores packed inside the worm (black arrow), bar = 10 μm. C) Higher magnification of myxospores revealing a typical *Myxidium *morphology, bar = 10 μm. A further figure plate of air-dried spores is available from the additional file 2 S2.

The infected monogenean from fish 2, after being flattened (Figure 1a), could be accurately identified as *Diplectanocotyla gracilis *Yamaguti, 1953. This identification was based on consistent features in the morphology and size of the haptoral sclerites as reported in Lim & Gibson [27] (all measurements in μm). Specifically, the two dorsal anchors lacking root processes, anchor base triangular, slightly crenulated; anchor inner length 31.5 (31-32), outer length 44 (43-45). Two ventral anchors with two prominent root processes; anchor inner length 37 (37), outer length 48.5 (48-49), outer root 6.5 (6.5) long and at 90° to the inner root 15 (15) long. The medial spine on the inner edge of the inner root of the ventral anchor, however, was not observed on either of the two ventral anchors, their position being obscured by the position of the ventral and dorsal bars overlying them. The two bifurcated dorsal bars measured 48 (46-50) long × 18 (17-19) wide, with a 13.5 (12-15) long, blunt-ended protrusion arising at a 45° (35°-55°) angle to the longitudinal axis of the 36 (32-40) long lanceolate portion of the bar. The two, open v-shaped ventral bars measured 51 (51) long, marked by a small, medial projection on the posterior edge; proximally lanceolate where they articulate with the ventral anchor; distal, medial orientated, ends of the two bars approximately triangular, the terminal part of which is slightly lobed.

### Description of myxosporean hyperparasite and host pathology

**Figure 2:**
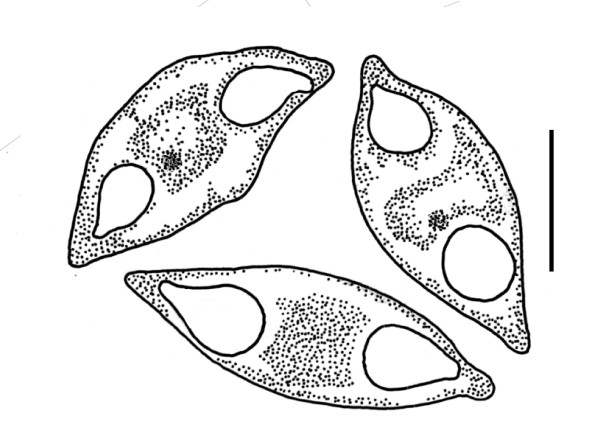
**Line drawings of three actual fresh spores of *Myxidium incomptavermi *n. sp. demonstrating minor variations observed between the morphologies**. Typically spores are fusiform with a slight sigmoid curve and pointed ends where opposing tear-shaped polar capsules are located. The polar filament had two, occasionally three turns (not shown) and a sporoplasm with a single nucleus was discernable between the polar capsules in some spores. The spore valves are smooth and the sutural line is inconspicuous. Bar = 5 μm.

Myxospore morphology was consistent with that of *Myxidium*, fusiform, sometimes slightly sigmoid, with pointed ends where opposing tear-shaped polar capsules are located (Figure 1b-c, Figure 2 & additional file 2, S2). The spore valves appeared smooth, without noticeable striations or ornamentation and the sutural line was inconspicuous. Mature spores (n = 20) measured 11.62 μm (11.27-11.75) in length, 4.92 μm (4.19-5.56) wide, polar capsule (PC) length 2.89 μm (2.43-3.27) PC width 1.96 (1.81-2.09). The polar filament had two, occasionally three turns and a sporoplasm with a single nucleus was discernable between the polar capsules in some spores. Development was histozoic in the parenchymal tissues of the monogenean host. Developing stages were not observed, but numerous paired spores were seen in the parenchymal tissues.

Infected monogeneans showed marked pathological changes, having poor internal structure definition, with areas of parenchymal tissues packed with myxospores (Figure 1a). The precise location of spores and extent of infection in the worms could not be fully determined due to the lack of internal structures in the specimens, but they were observed in the region anterior to the haptoral peduncle in the area occupied by the posterior vitellaria and the termini of the caecae. Histological and TEM data will assist future similar studies to answer outstanding questions about development and tissue location.

### Ribosomal DNA amplification and sequencing and phylogenetic analyses

The SSU rDNA for the hyperparasitic myxosporean was successfully amplified and sequenced from the single visibly infected monogenean from fish 1. Identical myxosporean DNA was also amplified and sequenced from the pooled gill monogeneans in seven out of 14 fish (50%), which included the two fish with visibly infected monogeneans. The contiguous novel myxosporean sequence of 1702 base pairs has been submitted to GenBank under the accession number GQ368246. A partial LSU rDNA sequence (922 bp) from the gill monogenean from fish 1 has also been deposited in GenBank under the accession number JN254760.

A nucleotide BLAST search of the novel myxosporean sequence revealed that the most similar sequences available in the databases are a *Myxidium *sp. hyperparasite sequence (GQ368245) isolated from gill monogeneans in Japan [14] with a 92% identity, followed by several *Kudoa *spp. isolated from marine fish, with similarities between 89-91%. The partial LSU rDNA sequence from the monogenean *D. gracilis *was most similar to *Diplectanum grouperi *(DQ054820) from the same family, Diplectanidae, with an 88% identity. The new myxosporean described from the parenchyma of *D. gracilis *ex *M. cyprinoides *is named as *Myxidium incomptavermi *n. sp.

**Figure 3:**
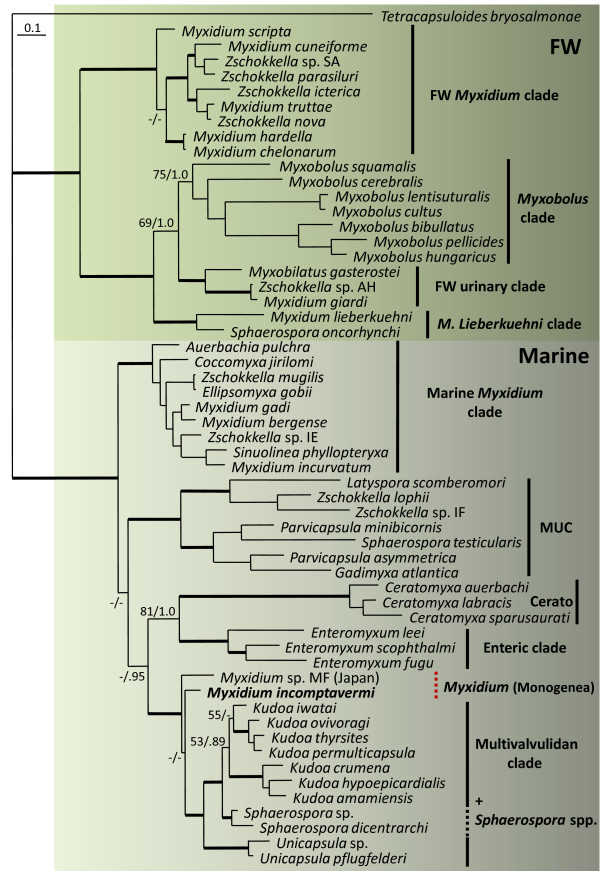
**Maximum likelihood phylogeny of myxozoan SSU rDNA sequence alignments of 57 taxa (2331 characters)**. Non-parametric bootstrap values and Bayesian inference posterior probabilities are shown at the nodes. Bold branches lead to a node with a high bootstrap support of ≥ 95 and a Bayesian posterior probability of ≥ 0.98. Nodes with a bootstrap support of < 50 and a Bayesian posterior probability of < 0.95 were considered not supported and are represented with a dash. The upper green boxed section contains freshwater (FW) species which includes 4 main clades: freshwater *Myxidium*, *Myxobolus*, urinary and *M. lieberkuehni*. The lower grey boxed section contains the marine species, which includes 5 main clades: marine *Myxidium*, marine urinary clade (MUC), *Ceratomyxa *(Cerato), enteric and multivalvulidan (including the bivalvulids *Sphaerospora *spp. (dashed line)), and the newly placed sequential lineages, *Myxidium *spp. from gill monogeneans (dashed red line) at the base of the multivalvulidan clade. Accession numbers for the taxa used can be found in additional file 1, S1.

Phylogenetic analyses of alignments of 57 myxozoan taxa using maximum likelihood and Bayesian inference analysis produced trees with the same basic topologies (Figure 3), apart from small differences in branch lengths and support values. *Myxidium incomptavermi *grouped towards the base of the multivalvulidan clade together with the monogenean hyperparasite sequence *Myxidium *sp. from Japan, and were both basal to the clade containing *Unicapsula *spp. The grouping of this clade was fully supported in both analyses (100/1.0) from the rest of the taxa in the tree. The placement of other myxosporean taxa and the overall tree topology was as expected, with *Myxidium *confirmed as being polyphyletic throughout the phylogeny in both the marine and freshwater groups.

### Myxosporean infections in the fish host

Examination of each gill arch from the 14 fish during monogenean removal revealed that no lesions or cysts were present on the gill filaments or cartilage. The only abnormality found was the presence of the copepod *Lernanthropus *sp. (family Lernanthropidae) on six of the 14 fish (43%). PCR of the gill filaments taken from the 14 fish, using the specific primer pair Bu-F/Bu-R, were negative, apart from a very faint band present in fish 2, which is known to have harboured infected monogeneans. Extensive tissue sampling was also performed on fish 2 during the final sampling trip and positive PCR results (good PCR bands at the expected size of 720 bp), using Bu-F/Bu-R, were obtained for kidney, spleen, intestine and stomach. Faint bands were just visible for gill, liver and brain; but gall bladder wall, bile contents and heart muscle were negative. The 720 bp amplicons from kidney, spleen, intestine and stomach were successfully sequenced and matched 100% with the myxosporean sequence obtained from infected gill monogeneans.

During fish dissections, no myxospores corresponding to the morphology of *M. incomptavermi *were found. However, two other myxosporeans, morphologically distinct from *M. incomptavermi*, were discovered, one from the kidney and one from the gall bladder. Partial SSU rDNA data has been obtained for these two myxosporeans, which are both dissimilar from that of *M. incomptavermi *and do not share phylogenetic affinities with the multivalvulidan group. These data will be published in a separate manuscript.

### Taxonomic summary and type material

Class Myxosporea Bütschli, 1881

Order Bivalvulida Shulman, 1959

Suborder Variisporina Lom et Noble, 1984

Family Myxidiidae Thélohan, 1892

Genus Myxidium Bütschli, 1882

*Myxidium incomptavermi *n. sp. (with morphological characters of the genus)

Type host (Monogenea): *Diplectanocotyla gracilis *Yamaguti, 1953

Fish host (Actinopterygii): *Megalops cyprinoides *(Broussonet, 1782), Indo-Pacific tarpon

Type locality: Coastal waters of Langkawi Island, Peninsular Malaysia

Site of infection: Parenchymal tissues of monogenean host. Detected by PCR only in certain fish tissues (kidney, spleen, intestinal tract)

Etymology (Latin): incompta refers to the poor morphological condition of heavily infected monogeneans

Type material: Two DiffQuik stained pertex mounted type slides of *Myxidium incomptavermi *n. sp. from *Diplectanocotyla gracilis *(Monogenea: Diplectanoidea) (ex *Megalops cyprinoides *Broussonet, 1782) collected from Langkawi, Malaysia have been deposited in the Natural History Museum (NHM), London with the following registration numbers (NHMUK) 2011.200 and 2011.201. In addition a CD of images of fresh and fixed spores has been deposited to the NHM collection.

## Discussion

### Spore morphology, development and identification

Myxospore morphology of *M. incomptavermi *is similar to that of the type species for the genus, *Myxidium lieberkuehni*. However, spores of *M. lieberkuehni *are larger (20 × 6 μm) with longitudinal striations and they are coelozoic in the renal system of the pike, *Esox lucius*, in Europe and North America [6]. *Myxidium giardi *has been described as hyperparasitic in a gill monogenean from the European eel, *A. anguilla*, but the spores are smaller (9 × 5.5 μm) than those of *M. incomptavermi *and have numerous prominent longitudinal ridges [28]. In addition, SSU rDNA sequence data exists for both *M. lieberkuehni *and *M. giardi*, confirming that they are different (Figure 3). The undescribed hyperparasitic *Myxidium *sp. from gill monogeneans of *Platycephalus *sp. in Japan [14] is phylogenetically related to *M. incomptavermi *(Figure 3), but the sequences are only 92% similar and they infect different hosts from different geographical locations. *Fabespora vermicola *and *Fabespora *sp. are myxosporean hyperparasites of the tegument and parenchymal tissues of digenean trematodes in brackish water sparid fish [11,12]. The spores of *Fabespora *spp. are morphologically distinct from those in the present study, having blunt ends with elongated valves perpendicular to a prominent central transverse sutural line.

Sporogony in *M. incomptavermi *may proceed in a similar manner to that of *M. lieberkuehni*, as paired spores can be seen inside infected worms [14], but when released no disporous plasmodia are found. In urinary bladder infections of *M. lieberkuehni*, numerous large plasmodia are found where pairs of spores are contained within a pansporoblast [28]. This type of development could take place in myxosporean-infected monogeneans, where large polysporous plasmodia form that contains paired spores. Indeed, unexplained large 'corpuscles' were described from *Myxidium*-infected gill monogeneans on the European eel by Aguilar et al. [13] that could represent such structures. However, pansporoblast formation has not been reliably observed during multivalvulidan sporogony [29] and histological studies will need to be undertaken on infected monogeneans to verify this possibility.

The vast majority of *Myxidium *spp. are coelozoic parasites, but *M. incomptavermi *is histozoic in the monogenean host. Molecular data has shown that *M. incomptavermi *is also present in certain fish tissues, however, as spores have never been observed it has not been possible to locate and evaluate its development within the fish host.

Due to its histozoic development within the monogenean host and differences in either spore morphology or rDNA sequence data to other *Myxidium *spp. infecting gill monogeneans, a new species, *M. incomptavermi *is described for this novel myxosporean hyperparasite.

### Prevalence and pathogenicity of *Myxidium incomptavermi*

Isolating visibly infected live gill monogeneans in the field was challenging and only two fish could be confirmed to harbour such worms. However, a subsequent PCR study of pooled monogenean DNA revealed that 50% of fish (7/14) had monogeneans with the myxosporean present. The majority of gill monogeneans (a sub-sample from heavily infected fish) were individually examined using a compound microscope, but in most cases infections with the myxosporean were not visibly detected. Heavily infected worms (Figure 1a) were relatively easy to identify as their appearance was noticeably different, but the vast majority of specimens examined microscopically appeared to be uninfected. From the PCR results it is clear that some of the monogeneans that were examined must have been infected with the myxosporean, suggesting that infections were probably at an early stage before spores had developed and any gross pathology was detectable. The relative lack of visibly infected monogeneans from those examined may indicate that the myxosporean causes serious pathology in the worm resulting in rapid mortality. However, the relative abundance of monogeneans from the two fish with observed hyperparasitism was not noticeably low (fish 1, 20 monogeneans collected; fish 2, 50 monogeneans collected) compared to other fish.

The relative scarcity of visibly myxosporean-infected platyhelminthes has also been noted in other studies. When *F. vermicola *was originally sampled, Overstreet [11] examined 71 fish from various Mississippi estuaries and only found the hyperparasite in three fish. Similarly, Aguilar et al. [13] only found hyperparasitism in one of 323 eels. But, what appears to be more notable is the number of infected individuals that were found in a fish that had the hyperparasite. Overstreet [11] examined 34 trematodes from a fish with infected worms and found 9 (26.5%) with mature spores, whilst Aguilar et al. [13] detected mature spores in 30% of the *P. bini *from the single eel and described unknown 'corpuscles' in 100% of *P. bini *sampled from same eel. Thus, it appears that although the overall prevalence of the hyperparasite may be extremely low, or hard to detect microscopically in a given fish population, the number of infected individuals from a fish with the hyperparasite present can be relatively high. In spite of the fact that myxosporean infections were not observed in the fish by Overstreet [11] or Aguilar et al. [13], the fish would appear to play a significant role in the presence of the infection in the worms in both cases.

In the present study, DNA from *M. incomptavermi *was indeed amplified from various fish tissues from a fish known to harbour a visibly infected monogenean. But, despite very careful examination of the same tissues, no myxospores of *M. incomptavermi *could be found in the fish. If gill monogeneans become infected by feeding on a myxosporean-infected fish then it would seem likely that the transfer of infection was via the gill epithelium. However, *D. gracilis *has not been reported to blood-feed [27] and no evidence of host blood or haematin was observed inside the monogeneans during this study, and gill filaments from the fish in this study were either negative or only very faintly positive, when tested with the specific PCR, especially when compared to other internal tissues. This suggests that the monogeneans do not become infected by simply feeding from the mucus and gill epithelia of an infected fish. It is possible that monogeneans might ingest sporoplasms that have been released from marine actinospores on the gill surface, before they enter the fish host. However, infection in the monogeneans is histozoic and not restricted to a superficial development in the anterior digestive organ where fish material would be present, if it was required for development to take place. In addition, myxospores are not detectable in the fish host implying that complete development may not occur in the fish, making it a dead-end host with no transmission potential for the myxosporean. Therefore, an alternative scenario could be that the fish has little to do with the infection or the transmission of the myxosporean parasite, but is simply being exposed to the same actinospore stages of this parasite. Occurrence in the blood-filtering tissues like kidney and spleen, as well as in the gastrointestinal tract supports this idea. The fish is exposed and the parasite can penetrate the integument, but development in the fish is stopped, the monogeneans simply become infected by grazing released sporoplasms on the gill surface.

Currently, the relationship between the fish and the monogenean and the dynamics of the myxosporean infection are not fully understood. Nevertheless, myxospores with a *Myxidium*-like morphology have been reported to develop in three different gill monogeneans infecting three different host fish (Table 1). DNA data exists for two of these myxosporean hyperparasites and they are phylogenetically related. In the third case, from European eels infected with two species of *Pseudodactylogyrus*, clear host specificity was observed, where one species, *P. bini*, was found to have a high prevalence of infection with the myxosporean and the other, *P. anguillae*, was completely uninfected. These facts confirm that gill monogeneans are true hosts for myxosporeans, but it remains to be clarified how this is related to infections in the fish.

**Table 1:** Hyperparasitic myxosporean infections of platyhelminthes

Myxosporean (reference)	Host platyhelminth	Host fish/habitat sampled from	Pathology to platyhelminth	**Developmental stages in platyhelminth**, Sporogony	Myxosporean observed in the fish host
*Myxidium incomptavermi* n. sp. (present study)	*Diplectanocotyla gracilis* gill monogenean	*Megalops cyprinoides* (Indo-Pacific tarpon) Brackish/mangroves Langkawi, Malaysia	Marked pathology, parenchymal tissues packed with spores.	Paired spores observed in parenchymal tissues. Likely polysporous within a pansporoblast	Not observed despite careful screening; but amplified via PCR
*Myxidium giardi* (doubtful ID) [13]	*Pseudodactylogyrus bini* gill monogenean	*Anguilla anguilla* (European eel) River in Northwest Spain.	Not mentioned, but numerous 'corpuscles' and spores are always present, replacing monogenean tissues.	Not described, but corpuscles of differing sizes were present and only in hyperparasitised individuals.	*Myxidium giardi *was not found in the eel with hyperparasitic myxosporeans
*Myxidium *sp. [14]	*Haliotrema *sp. gill monogenean	*Platycephalus *sp. (flathead) Brackish water, Lake Hamana, Japan	Marked pathology, parenchymal tissues packed with spores.	Paired spores observed in parenchymal tissues. Likely polysporous within a pansporoblast	Not found, despite careful screening
*Fabespora vermicola* [11]	*Crassicutis archosargi* intestinal digenean	*Archosargus probatocephalus* (sheepshead seabream) Estuarine, East USA.	Highly pathogenic, necrosis of the gonads leads to a total loss of fecundity.	Histozoic trophozoites, in parenchymal cells and in the integument. Disporous.	Not found, despite careful screening
*Fabespora *sp. [12]	*Allopodocotyle chrysophrii* intestinal digenean	*Sparus aurata* (gilthead seabream) Brackish water lagoon and Mediterranean coast	Sporogonic stages present within the tegument and parenchymal tissues cause tissue damage.	Monosporous in the tegument, and generally disporous in the parenchymal tissues.	Not found

### Molecular phylogenetics

The SSU rDNA of *M. incomptavermi *was relatively short at 1702 bases, but is consistent with phylogenetically related multivalvulidan taxa. In the phylogenetic analyses, irrespective of the tree-building methodology used, *M. incomptavermi *is consistently and robustly located at the base of the multivalvulidan clade together with the hyperparasitic *Myxidium *sp. from gill monogeneans from Japan.

Although support for the grouping of the multivalvulidan clade was very robust in both analyses (100/1.0), *M. incomptavermi *occupies a single poorly supported branch in the trees. Therefore, additional sequence data from other hyperparasitc myxosporeans, such as *Fabespora*, may help to confirm this location at the base of the multivalvulidan clade, and alternative gene data for *M. incomptavermi *and other bivalvulid taxa in the multivalvulidan group, such as certain *Sphaerospora *spp., will help to clarify the evolution of the Kudoidae myxospore form.

### *Myxidium giardi *as a hyperparasite of eel monogeneans

Aguilar et al. [13] reported the presence of *Myxidium *spores in the gill monogenean *P. bini *from the European eel *A. anguilla *in Spain, and concluded that it was *M. giardi *as this is a common gill myxosporean found in *A. anguilla*. In their paper, it is not possible to determine detailed spore morphology from the figures, however the spores they show in Figure 1C appear to be more bluntly rounded and are approximately L 6 × W 3.5 μm and not spindle-shaped and L 9 × W 5.5 μm as described for *M. giardi *[28]. From 323 eels examined, they only found the hyperparasitic *Myxidium *in gill monogeneans from a single eel, where myxospores were observed in 30% of *P. bini *individuals but were absent from *P. anguillae *present on the gills of the same eel. In addition, *M. giardi*, which had a 95% prevalence amongst the eels sampled, was not detected in the eel with the hyperparasitic *Myxidium*. Aguilar et al. [13] also described unknown, variable sized objects (40-265 μm) they termed 'corpuscles' that were present inside all *P. bini *samples from the single eel that harboured the hyperparasite, but were absent from all other specimens of *P. bini *from the 322 other eels and were also absent in *P. anguillae *from the eel with hyperparasites. As no myxospores were observed in the corpuscles they concluded that they were probably not parasitic in origin, but clearly had a strong association with the hyperparasitic condition in *P. bini*. We believe that the *Myxidium *spores observed in *P. bini *were most likely not *M. giardi*, but an unknown *Myxidium *myxosporean infecting *P. bini*. Furthermore, we suggest that the corpuscles are a developmental stage that could represent large developing plasmodia or pansporoblasts that will mature to contain numerous, potentially paired, spores. In addition, this *Myxidium *sp. has shown strong host specificity as no *P. anguillae *individuals were infected in the same eel, suggesting that it is most likely not an accidental infection of *M. giardi *in gill monogeneans.

*Myxidium giardi *is currently the only species of *Myxidium*, from over 200 described species, where the life cycle has been experimentally demonstrated, having aurantiactinomyxon-type actinospores in an oligochaete host [30], confirming that gill monogeneans are not a typical host and not required in the life cycle. Furthermore, in the phylogenetic analyses, *M. giardi *is robustly positioned in the freshwater urinary clade (Figure 3) and is phylogenetically distant to the *Myxidium*-like hyperparasites of monogeneans that group at the base of the multivalvulidan clade.

### Similarities in myxosporean hyperparasitism of platyhelminths and platyhelminths as hosts for the Myxozoa

Some interesting aspects appear to be shared among the five examples of myxosporean hyperparasitism of platyhelminths known to date (Table 1). Firstly, the fact that all the fish hosts studied (*Anguilla*, *Archosargus*, *Megalops*, *Platycephalus*, and *Sparus*) were sampled from coastal, brackish or estuarine environments. Secondly, that myxospores of the same morphology have never been observed in any of the fish hosts that harbour the infected platyhelminths. Thirdly, evidence of myxosporean development exists in all examples and subsequent pathological changes in the worms are reported in all cases.

As brackish water environments represent the transition between freshwater and the oceans, it remains possible that fish platyhelminths have somehow been involved in the radiation of the Myxozoa between the two environments, and currently available DNA data does place the known myxosporeans of monogeneans as basal lineages of one of the main marine clades. In evolutionary terms, the brackish water environment can be seen as a key location where myxosporeans established oligochaete worms as hosts and facilitated their subsequent radiation into freshwater [31]. Therefore, it is possible that other such evolutionary significant events occurred in this transitional environment between marine and freshwater.

It is odd that myxospores have never been isolated from the fish hosts in all known cases to date (Table 1), but can be found in relatively large numbers in heavily infected platyhelminths. It is possible that sites of infection in fish were simply overlooked or that mature spores rarely develop and the infection is somewhat latent. In contrast, in the worms, serious pathology results, which could suggest a more recent association. Overstreet [11] considered that *F. vermicola *had evolved from a piscine-inhabiting ancestor but again despite numerous attempts was never successful in finding the myxosporean parasite in the fish host.

Myxosporeans are known to infect an extremely wide range of both vertebrate and invertebrate groups. Therefore, it is not surprising that monogeneans and other platyhelminth parasites of fish can also act as hosts. The life cycles of marine myxosporeans are under represented compared to the freshwater group, and little or no data on invertebrate or non-fish hosts are currently available for the majority of genera with no data available for those of the Multivalvulida. In known marine myxosporean life cycles, the fish host harbours the myxospore stage and the invertebrate host the actinospore stage. Complete development of the hyperparasitic *Myxidium *may occur within the platyhelminth or another invertebrate may be required.

## Conclusions

Heavy myxosporean infections causing serious pathology in the gill monogenean, *D. gracilis *were rarely observed. But sub-clinical infections, only detectable using PCR, were found in monogeneans from 50% of fish sampled. Morphology of mature spores in heavily infected worms was typical of *Myxidium*, but development was histozoic in the parenchymal tissues of the monogenean. Simultaneous infections in the fish could not be confirmed microscopically despite extensive dissections; however, the parasite DNA could be amplified from kidney, spleen, intestine and stomach samples from fish known to harbour heavily infected monogeneans, suggesting that the fish could be involved in the life cycle of the myxosporean. However, it is also possible that fish are accidental or dead-end hosts and have little to do with the transmission of the myxosporean and are simply exposed to the same actinospores in similar quantities. This could result in the successful penetration of the sporoplasm into fish tissues; hence the PCR detection in blood-filtering and excretory tissues, but no resulting infection or myxospore development occurs. The later scenario may suggest that the monogenean has more recently evolved as a host for the myxosporean parasite, and the serious pathology observed in infected platyhelminths would support this theory of a more recent association.

SSU rDNA for the hyperparasite was successfully amplified and found to be most similar to another hyperparasitic *Myxidium *sp. also isolated from gill monogeneans. Phylogenetic analyses robustly placed both of these hyperparasitic myxosporean sequences at the base of the marine multivalvulidan clade. Although *Myxidium *spp. are known to be distributed in a polyphyletic manner throughout myxosporean phylogenetics, they are currently absent from the multivalvulidan order. Therefore, it is very interesting that the *Myxidium *parasites from gill monogeneans have been phylogenetically placed as sequential taxa, basally to the multivalvulidan order in our analyses. This suggests that the Multivalvulida may have radiated from a *Myxidium *spore form.

The true relationships between the myxosporean infections in gill monogeneans and host fish are not yet fully understood. However, myxospores with a *Myxidium*-like morphology, some of which we have shown to be phylogenetically related, have now been reported to develop in three different monogeneans. This suggests that these myxosporeans are true parasites of gill monogeneans and not just accidental infections of fish-infecting species.

Due to the histozoic development and the novel monogenean host, a new species, *Myxidium incomptavermi *is described for this myxosporean hyperparasite.

## Competing interests

The authors declare that they have no competing interests.

## Authors' contributions

MAF sampled the fish, isolated the gill monogeneans, photographed and measured the fresh myxospores. APS identified the monogeneans and made line drawing of the myxospores. MAF performed the molecular study and drafted the manuscript. Both authors read and approved the final version of the manuscript.

## Supplementary Material

Additional file 1 **Supplementary data (S1)**. 56 additional myxosporean taxa used in the phylogenetic analyses with accession numbers. Click here for file

Additional file 2 **Supplementary data (S2)**. Air-dried unstained spores of *Myxidium incomptavermi *n. sp., bar = 10 μm. Click here for file
