# Supplementary material for: Myxosporean hyperparasites of gill monogeneans are basal to the Multivalvulida
Source: Parasit Vectors. 2011 Nov 24;4:220. doi: 10.1186/1756-3305-4-220 (PMC3235069; doi:10.1186/1756-3305-4-220)
Supplement: Additional file 1 — Supplementary data (S1). 56 additional myxosporean taxa used in the phylogenetic analyses with accession numbers. [file 1756-3305-4-220-S1.PDF]

Supplementary data (S1)

56 additional myxosporean taxa used in the phylogenetic analyses with accession numbers

Taxa	accession number	Taxa	accession number
Auerbachia pulchra	DQ377703	Myxidum lieberkuehni	X76638
Ceratomyxa auerbachi	EU616732	Myxobilatus gasterostei	AY495703
Ceratomyxa labracis	AF411472	Myxobolus bibullatus	AF378336
Ceratomyxa sparusaurati	AF411471	Myxobolus cerebralis	U96492
Coccomyxa jirilomi	DQ323044	Myxobolus cultus	AB121146
Ellipsomyxa gobii	AY505127	Myxobolus hungaricus	AF448444
Enteromyxum fugu	AY520573	Myxobolus lentisuturalis	AY119688
Enteromyxum leei	AY520574	Myxobolus pellicides	AF378339
Enteromyxum scophthalmi	AF411335	Myxobolus squamalis	U96495
Gadimyxa atlantica	EU163418	Parvicapsula asymmetrica	AY584191
Kudoa amamiensis	AF034638	Parvicapsula minibicornis	AF201375
Kudoa crumena	AF378347	Sinuolinea phyllopteryxa	DQ645952
Kudoa hypoepicardialis	AY302722	Sphaerospora dicentrarchi	AY278564
Kudoa iwatai	AY514039	Sphaerospora oncorhynchi	AF201373
Kudoa ovivoragi	AY152750	Sphaerospora sp. IF-2006	DQ377695
Kudoa permulticapsula	AY078429	Sphaerospora testicularis	HM230825
Kudoa thyrsites	AY152747	Tetracapsuloides bryosalmonae	U70623
Latyspora scomberomori	HM230826	Unicapsula pflugfelderi	AM931470
Myxidium bergense	DQ377702	Unicapsula sp.	AY302725
Myxidium chelonarum	DQ377694	Zschokkella icterica	DQ333434
Myxidium cuneiforme	DQ377709	Zschokkella lophii	DQ301509
Myxidium gadi	DQ377711	Zschokkella mugilis	AF411336
Myxidium giardi	AJ582213	Zschokkella nova	DQ377688
Myxidium hardella	AY688957	Zschokkella parasiluri	DQ377689
Myxidium incurvatum	DQ377708	Zschokkella sp. AH -2003	AJ581918
Myxidium scripta	DQ851568	Zschokkella sp. IE -2005	DQ452716
Myxidium truttae	AF201374	Zschokkella sp. SA -2005	DQ118776
Myxidium sp. MF-2010	GQ368245	Zschokkella sp. IF -2006	DQ377705
