# Supplementary material for: Myxosporean hyperparasites of gill monogeneans are basal to the Multivalvulida
Source: Parasit Vectors. 2011 Nov 24;4:220. doi: 10.1186/1756-3305-4-220 (PMC3235069; doi:10.1186/1756-3305-4-220)

Supplementary data (S2)

Air-dried unstained spores of *Myxidium incomptavermi* n. sp.,
bar = 10 µm

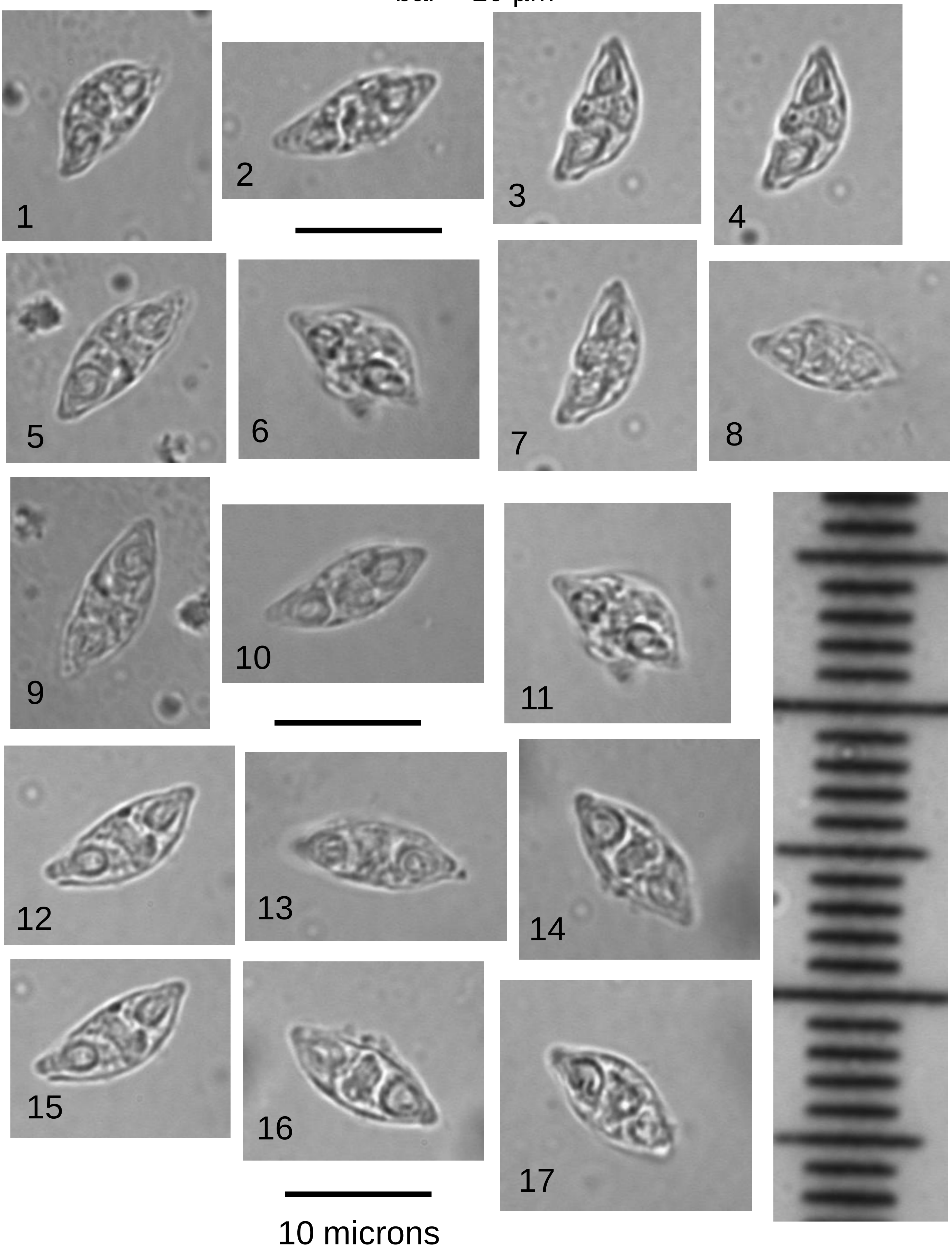

Supplement: Additional file 2 — Supplementary data (S2). Air-dried unstained spores of Myxidium incomptavermi n. sp., bar = 10 μm. [file 1756-3305-4-220-S2.PDF]
